# PredCRP: predicting and analysing the regulatory roles of CRP from its binding sites in *Escherichia coli*

**DOI:** 10.1038/s41598-017-18648-5

**Published:** 2018-01-17

**Authors:** Ming-Ju Tsai, Jyun-Rong Wang, Chi-Dung Yang, Kuo-Ching Kao, Wen-Lin Huang, Hsi-Yuan Huang, Ching-Ping Tseng, Hsien-Da Huang, Shinn-Ying Ho

**Affiliations:** 1 Institute of Bioinformatics and Systems Biology, National Chiao Tung University, Hsinchu, Taiwan; 2 Department of Biological Science and Technology, National Chiao Tung University, Hsinchu, Taiwan; 3 Institute of Population Health Sciences, National Health Research Institutes, Miaoli, Taiwan; 4 Department and Institute of Industrial Engineering and Management, Minghsin University of Science and Technology, Hsinchu, Taiwan; 5 Department of Laboratory Medicine, China Medical University Hospital, Taichung, Taiwan

## Abstract

Cyclic AMP receptor protein (CRP), a global regulator in *Escherichia coli*, regulates more than 180 genes via two roles: activation and repression. Few methods are available for predicting the regulatory roles from the binding sites of transcription factors. This work proposes an accurate method PredCRP to derive an optimised model (named PredCRP-model) and a set of four interpretable rules (named PredCRP-ruleset) for predicting and analysing the regulatory roles of CRP from sequences of CRP-binding sites. A dataset consisting of 169 CRP-binding sites with regulatory roles strongly supported by evidence was compiled. The PredCRP-model, using 12 informative features of CRP-binding sites, and cooperating with a support vector machine achieved a training and test accuracy of 0.98 and 0.93, respectively. PredCRP-ruleset has two activation rules and two repression rules derived using the 12 features and the decision tree method C4.5. This work further screened and identified 23 previously unobserved regulatory interactions in *Escherichia coli*. Using quantitative PCR for validation, PredCRP-model and PredCRP-ruleset achieved a test accuracy of 0.96 (=22/23) and 0.91 (=21/23), respectively. The proposed method is suitable for designing predictors for regulatory roles of all global regulators in *Escherichia coli*. PredCRP can be accessed at https://github.com/NctuICLab/PredCRP.

## Introduction

Cyclic AMP receptor protein (CRP) is one of the most important transcription factors (TFs) in *Escherichia coli* (*E. coli*). CRP was the first purified^1^, crystallised^2^, and the most well-studied TF in *E. coli*^3–9^. CRP regulates more than 180 genes^10^ by cooperating with cAMP. The latter transduces intracellular signals by changing its intracellular concentration in response to environmental signals. Once the concentration of intracellular cAMP changes, the production of the cAMP-CRP complex will be affected^10–14^. When the cAMP-CRP complex acts on a binding site for gene regulation, it has one of two opposing regulatory roles: activation or repression^15^. The CRP-regulated genes are typically involved in energy-related metabolic pathways, such as galactose metabolism, citrate metabolism, and the phosphoenolpyruvate group translocation system^16^.

To predict the regulatory roles of CRP, we first needed to know more about the CRP-binding site. There are two categories of methods to identify the binding site of a specific TF. One category consists of the low-throughput methods such as the electrophoretic mobility shift assay and the DNase I footprinting assay^17^. The other category comprise high-throughput methods such as the chromatin immunoprecipitation (ChIP) assay, a sequencing-based method (ChIP-seq)^18^ and elegant computational methods focusing on predicting TF-binding sites^19–21^. Once information on the CRP-binding sites has been obtained, we can further predict the regulatory roles of CRP.

There are two approaches to determining regulatory roles of a TF. One is a low-throughput approach such as the promoter*-lacZ* fusion^17^ and quantitative PCR (qPCR)^22^. The other consists of a high-throughput approach such as gene expression analysis using RNA-seq^23^ or microarray data. However, gene expression analysis has a limit in determining indirect effects such as co-expressed but not co-regulated genes^24^. Currently, the identification of directly co-regulated genes is particularly challenging^24^. These observations imply that the determination of the regulatory roles of a TF on co-regulated genes is also a challenging problem.

Few methods are available for predicting the regulatory roles of a global TF from TF-binding sequences. The widely used 3-class rules state that if a CRP-binding site satisfies some conditions, CRP tends to be an activator. The Class I rule states that if the CRP-binding site is located at position −61.5, then CRP tends to be an activator^25–27^. The Class II rule states that if the CRP-binding site is located at position −41.5, then CRP tends to be an activator^25–27^. The Class III rule states that if a CRP-dependent promoter has two or more CRP-binding sites, then the promoter tends to be an activator^25,27^. At present, there is no rule linking conditions in the cAMP-CRP complex to it acting as a repressor^25–27^.

**Figure 1:**
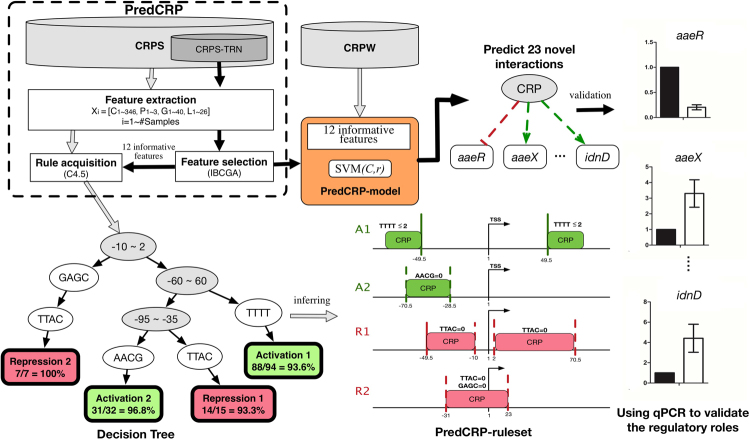
Components for developing and evaluating the proposed PredCRP method. (1) establishment of the CRPS dataset consisting of 169 CRP-binding sites with regulatory roles supported by strong evidence, (2) feature extraction from the training dataset CPRS-TRN, (3) feature selection in cooperation with an SVM, (4) PredCRP-ruleset obtained based on the decision tree method C4.5, (5) PredCRP-model evaluated by CRP-binding sties with weak evidence (the CRPW dataset) and (6) the qPCR experimental validation on the regulatory roles of CRP.

Hence, we propose an accurate method, PredCRP, to derive an optimised model (named PredCRP-model) and a set of four interpretable rules (named PredCRP-ruleset) for predicting and analysing the regulatory roles of CRP from given sequences of CRP-binding sites. The design of PredCRP includes four parts (see Fig. 1): (1) establishment of a dataset consisting of 169 CRP-binding sites with regulatory roles strongly supported by evidence, (2) feature extraction from the CRP-binding sites, (3) feature selection in cooperation with a support vector machine (SVM), and (4) rule acquisition based on the decision tree method C4.5. More information about feature extraction, feature selection, and rule acquisition are found in the Materials and Methods section.

As a result, a set of 12 informative features was identified from 380 candidate features of CRP-binding sites using an inheritable bi-objective combinatorial genetic algorithm (IBCGA)^28^ (see the Materials and Methods section). Various prediction models were evaluated to examine both prediction accuracy and interpretation ability. PredCRP-model achieved a training and test accuracy of 0.98 and 0.93, respectively. PredCRP-ruleset covered 88% of the CRP-binding sites and achieved a training accuracy of 0.95. Among the four interpretable rules, the covered binding regions of two activation rules contain the regions of the widely used Class I and II rules of CRP. The other two rules for the repression role are novel and describe the condition relating to the CRP-binding site’s locations and sequence composition. Furthermore, we used PredCRP-model and PredCRP-ruleset to screen and identify 23 previously unobserved regulatory interactions in *E. coli* and validated these regulatory roles using qPCR experiments. Experimental results showed that 22 and 21 of the 23 interactions were correct using PredCRP-model and PredCRP-ruleset, respectively.

## Results

### Evaluation of PredCRP-model

Twelve informative features were selected using the IBCGA, which include eight of the 256 4-mer motifs in the composition descriptor (AACG, CATT, GAAC, GAGC, TGCG, TTAC, TTAT, and TTTT) and four features in the location-dependent descriptor: (1) L3: the size of the overlap region between the CRP-binding site and the region from −35 to −10; (2) L6: the size of the overlap region between the CRP-binding site and the region from −10 to 2; (3) L12: the size of the overlap region between the CRP-binding site and the region from −60 to 60; and (4) L15: the size of the overlap region between the CRP-binding site and the region from −95 to −35.

Tables 1 and 2 show the performance comparison among various feature sets with the SVM classifier on the training (CRPS-TRN) and test (CRPS-TST) datasets, respectively. The parameter settings of the SVMs for various feature sets except for PredCRP-model were determined by a grid search method. The positive and negative sites are the binding sites on which CRP act as a repressor and an activator, respectively. The all-feature SVM model works as a baseline model. PredCRP-model with the 12 informative features was significantly better than SVMs with various feature sets for both the training and test datasets (Tables 1 and 2). PredCRP-model achieved test ACC and MCC values of 0.93 and 0.79, respectively. The feature selection of PredCRP enhanced the SVM classifier with all features by test ACC 0.13 (=0.93–0.80) and MCC 0.53 (=0.79–0.26). The additional dataset (named CRPS-TST-2) was used to evaluate PredCRP-model in this study. PredCRP-model achieved test ACC and MCC values of 0.97 and 0.91, respectively.

**Table 1:** Prediction performance comparisons between PredCRP-model and the SVM-based methods with various feature sets on the CRPS-TRN dataset.

Feature set	No. of features	SPE	SEN	MCC	ACC
PredCRP-model	12	1.00	0.92	0.95	0.98
Informative 4-mer motifs	8	0.99	0.38	0.52	0.86
Informative location features	4	1.00	0.29	0.49	0.85
All features (baseline)	380	0.98	0.29	0.41	0.83
Composition descriptor	320	0.98	0.17	0.26	0.81
Location-dependent descriptor	17	0.99	0.29	0.45	0.84
Location-independent descriptor	363	0.99	0.17	0.31	0.81

**Table 2:** Prediction performance comparisons between PredCRP-model and the SVM-based method with various feature sets on the CRPS-TST dataset.

Feature set	No. of features	SPE	SEN	MCC	ACC
PredCRP-model	12	0.95	0.83	0.79	0.93
Informative 4-mer motifs	8	0.97	0.25	0.36	0.82
Informative location features	4	0.98	0.25	0.36	0.82
All features (baseline)	380	0.98	0.17	0.26	0.80
Composition descriptor	320	0.93	0.33	0.33	0.80
Location-dependent descriptor	17	1.00	0.08	0.26	0.80
Location-independent descriptor	363	0.93	0.33	0.33	0.80

**Figure 2:**
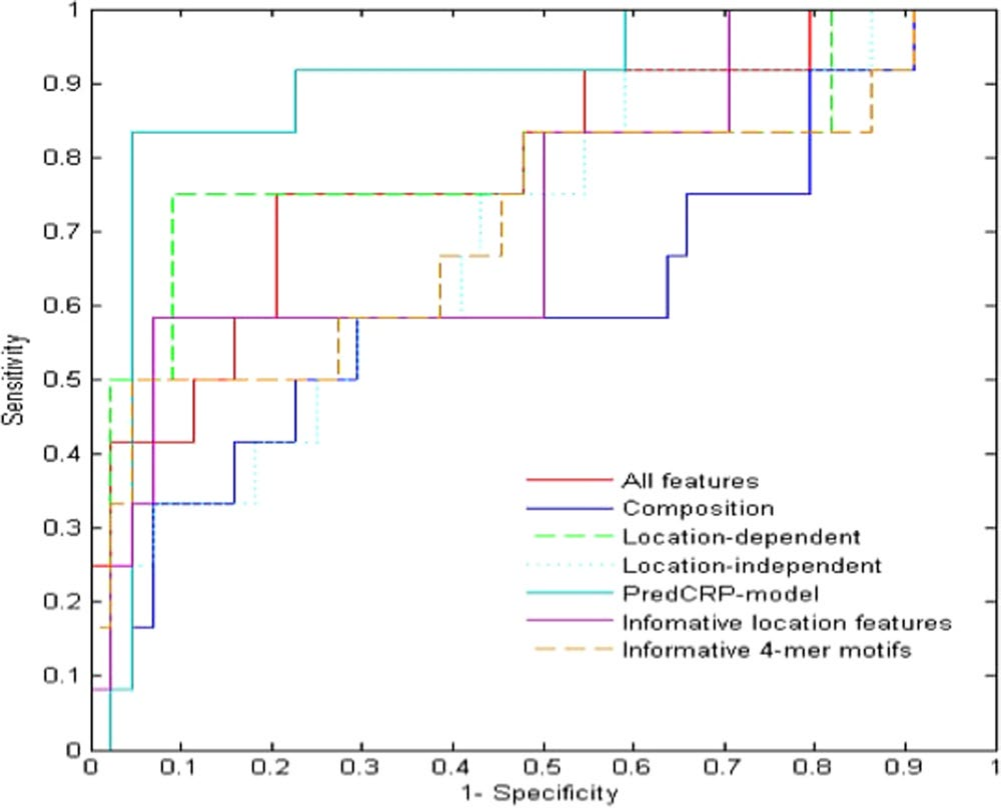
ROC curves of various methods using the CRPS-TST dataset. The AUCs of PredCRP-model and SVMs with informative 4-mer motifs, informative location features, all features, composition feature, location-dependent feature, and location-independent feature were 0.71, 0.73, 0.90, 0.79, 0.61, 0.79, and 0.70, respectively.

To avoid threshold setting bias, we further compared performance in terms of the area under the receiver operating characteristic (ROC) curve (AUC). As shown in Fig. 2, PredCRP-model has the AUC value of 0.90 with the test dataset, which is better than the SVM method with other feature sets: 0.71 for eight informative 4-mer motifs, 0.73 for four informative location features, 0.79 for all features, 0.61 for composition features, 0.79 for location-dependent features, and 0.70 for location-independent features.

The SVMs with the eight motif features and four location features yielded test accuracy of 0.82 and 0.82, respectively. This result reveals that the use of the 4-mer motifs or location features only cannot produce satisfactory prediction results. Furthermore, the use of a combination of informative motif and location features is important for accurate prediction. PredCRP-model makes the best use of both the informative 4-mer motifs and location features.

### Evaluation of PredCRP-ruleset

**Figure 3:**
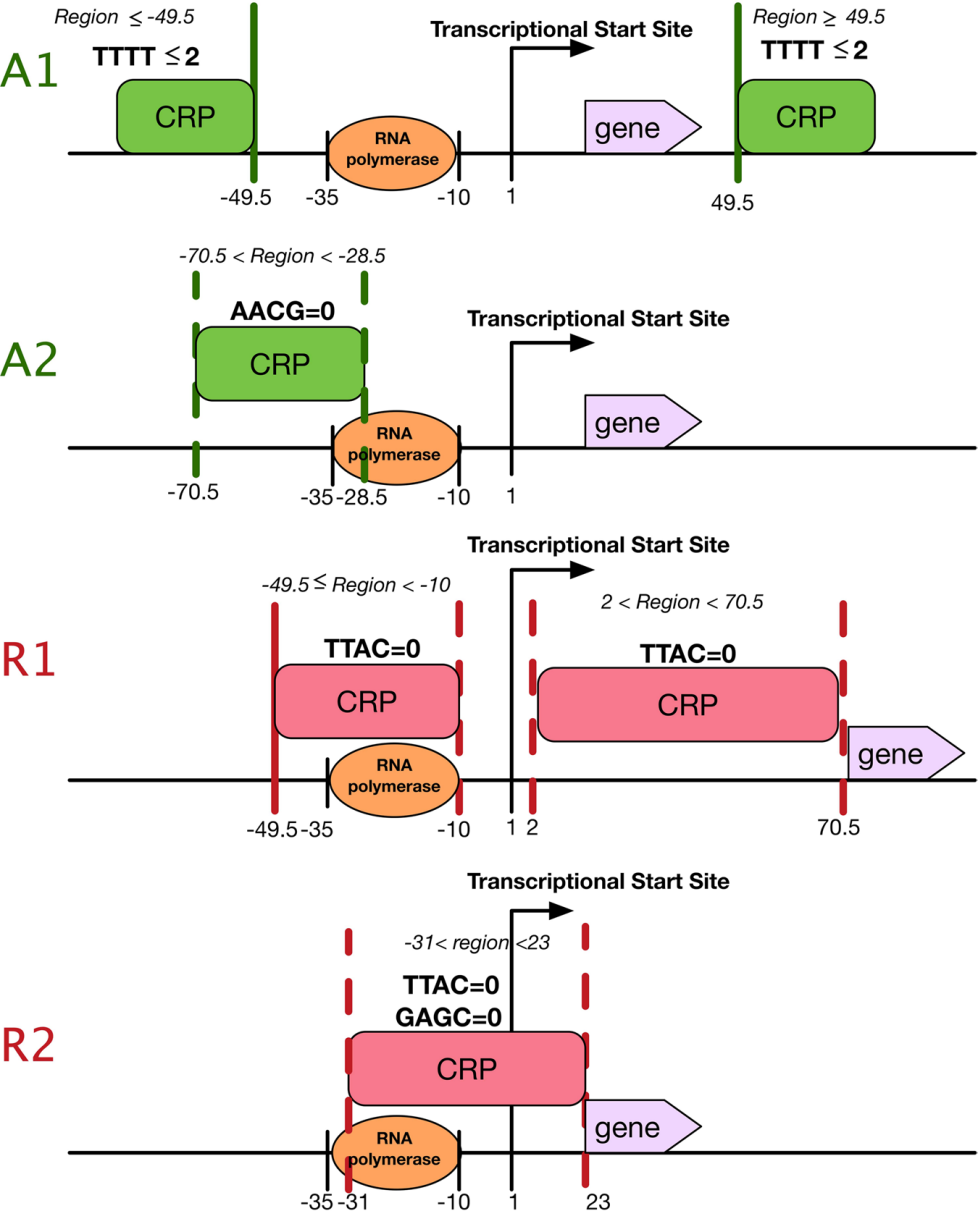
Four interpretable rules for illustrating the regulatory roles of CRP acting on the binding region.

PredCRP-ruleset has two activation rules and two repression rules obtained from the decision tree method C4.5, along with the 12 informative features. Each extracted interpretable rule corresponds to a specific path from the root to a specific leaf of the decision tree. Each rule has its own cover ratio on the CRPS dataset consisting of 133 activators and 36 repressors. Figure S1 shows the extracted rules for activators and repressors. After careful inference of the location and motif features, two activation rules and two repression rules with high cover ratios were derived, as shown in Fig. 3. The detailed inference procedure is presented in Supporting Information, and the location criteria of activation rules 1 and 2, and repression rules 1 and 2 are shown in the supplementary figures (Figures S2, S3, S4, and S5).

#### Activation rule 1

The activation rule 1 (A1) states that if a CRP-binding site is located in binding regions denoted as location variable *Region*, where *Region* ≤ −49.5 and *Region* ≥ 49.5, and the number of TTTT motifs is smaller than or equal to 2 in a CRP-binding site, then CRP generally acts as an activator. A1 covers 66.2% (=88/133) of activators in the CRPS dataset and shows an accuracy of 93.6% (=88/94). Furthermore, the binding regions of A1 contain the region of the widely used Class II rule of CRP. The *Region* ≤ −49.5 of A1 covers 87 activators and the *Region* ≥ 49.5 covers only one activator.

#### Activation rule 2

The activation rule 2 (A2) states that if a CRP-binding site is located in the binding region of −70.5 to −28.5 and there is no AACG motif in the CRP-binding site, then CRP generally acts as an activator. A2 covers 23.3% (=31/133) of activators in the CRPS dataset and shows accuracy of 96.8% (=31/32). Furthermore, the binding region of A2 contains the region of the widely used Class I rule of CRP.

#### Repression rule 1

The repression rule 1 (R1) states that if a CRP-binding site is located in the binding regions, where 2 < *Region* <70.5 and −49.5 ≤ *Region* <−10, and there is no TTAC motif in the CRP-binding site, then CRP generally acts as a repressor. R1 covers 38.9% (=14/36) of repressors in the CRPS dataset and shows accuracy of 93.3% (=14/15). The binding region (−49.5 to −10) strongly overlaps with the RNA polymerase-binding site (−35 to −10). On the other hand, the binding region (2 to 70.5) is located downstream of the transcription start site. Therefore, when CRP binds to these regions, it may block the transcription process. The 2 < *Region* <70.5 of R1 covers five repressors, and the −49.5 ≤ *Region* <−10 covers nine repressors.

#### Repression rule 2

The repression rule 2 (R2) states that if a CRP-binding site is located in the binding region of −31 to 23 and if neither TTAC nor GAGC motif is present in this CRP-binding site, then CRP generally acts as a repressor. R2 covers 19.4% (=7/36) of repressors in the CRPS dataset and shows accuracy of 100% (=7/7). This binding region of −31 to 23 also strongly overlaps with the RNA polymerase-binding site (−35 to −10). Hence, when CRP binds to the region (−31 to 23), it may block the transcription process.

### Validation of the predicted regulatory roles of CRP by quantitative PCR experiments

**Figure 4:**
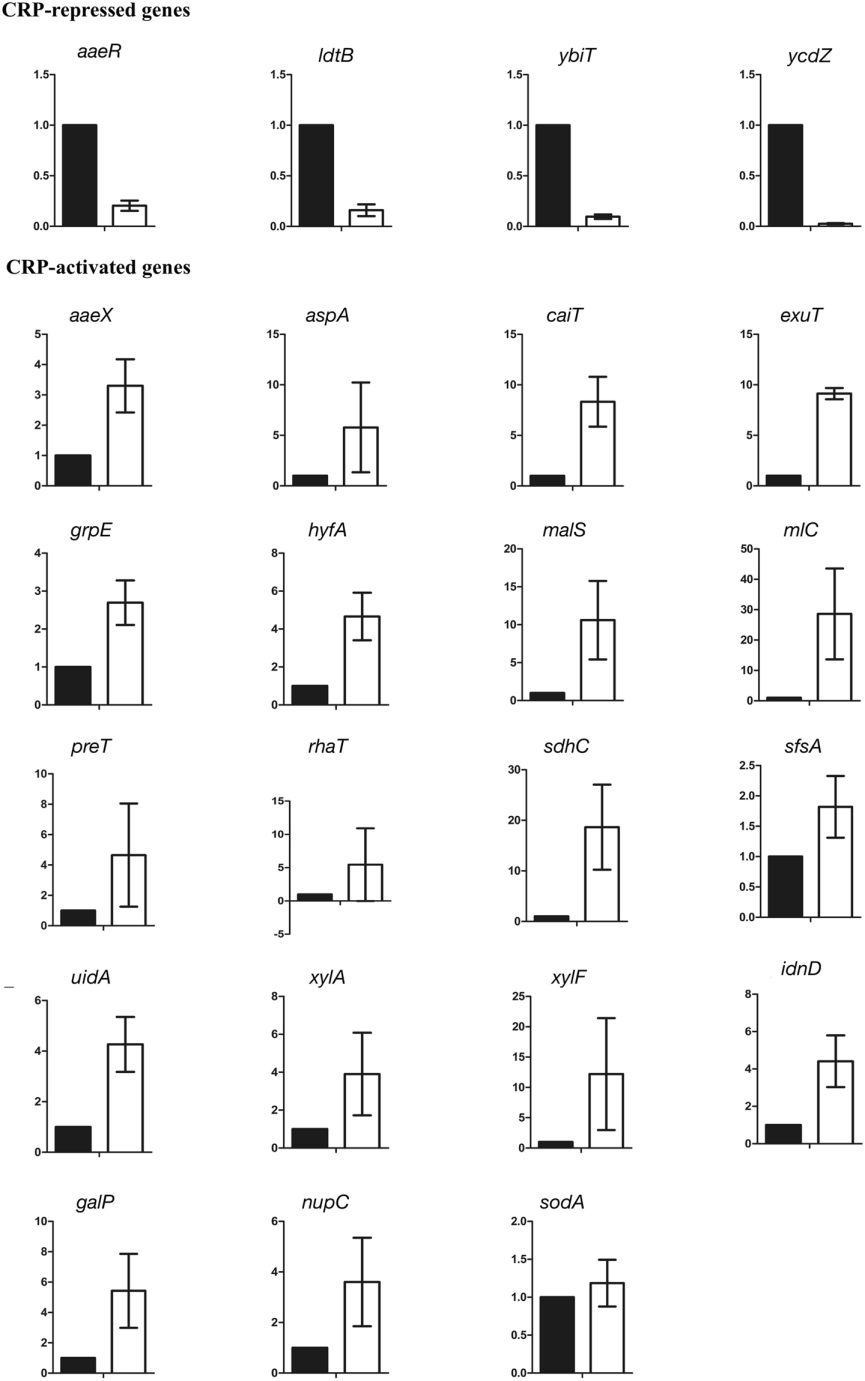
The quantitative PCR experiments for determining the regulatory roles of CRP on the 23 previously unobserved regulatory interactions in *E. coli*. The y-axis represents the relative quantity. The whiskers are the standard deviation of relative quantity. The black bars belong to the control group (0 mM cAMP concentration), and the white bars belong to the case group (1 mM cAMP concentration).

This work utilised the interpretable rules to predict the regulatory roles of CRP acting on 23 CRP-binding sites with weak evidence. The predicted roles of CRP were validated by quantitative PCR experiments. The results show that the prediction accuracy of PredCRP-model is as high as 0.96 (=22/23). The only wrong prediction of CRP-binding sites occurred on the *ldtB* gene in which CRP acts as a repressor based on qPCR validation, but PredCRP-model predicted an activator role. On the other hand, the prediction accuracy of the PredCRP-ruleset is 0.91 (=21/23). The wrong prediction of CRP-binding sites occurred on the *ldtB* and *exuT* genes. Based on the qPCR validation, the regulatory roles of CRP acting on the *ldtB* and *exuT* genes are repressor and activator, respectively, which PredCRP-ruleset predicted to have opposite roles. The results of the qPCR experiments are shown in Fig. 4 and Table S2, and the inference of relative quantity in qPCR is in the Supporting Information.

## Discussion

Considering the trade-off between prediction accuracy and interpretation ability, both an SVM model and a set of interpretable rules were proposed. PredCRP-model had a higher accuracy of 0.96 (=22/23) but with less interpretation ability. PredCRP-ruleset obtained satisfactory accuracy of 0.91 (=21/23) and also provides a cover ratio that biologists can easily analyse for the regulatory roles of CRP-binding sites.

The regulatory role of CRP acting on *ldtB* was wrongly predicted using PredCRP-model. On the other hand, the regulatory roles of CRP acting on both *ldtB* and *exuT* were wrongly predicted using PredCRP-ruleset. The CRP-binding site of *ldtB* has no informative 4-mer motifs (AACG, CATT, GAAC, GAGC, TGCG, TTAC, TTAT, or TTTT), which satisfies the motif conditions of the four rules. Nonetheless, its location satisfies the A2 rule only. The *ldtB* gene is annotated with the following GO terms: GO:0006508 (proteolysis), GO:0009252 (peptidoglycan biosynthetic process), GO:0043164 (gram-negative-bacterium-type cell wall biogenesis), and GO:0030288 (outer membrane-bounded periplasmic space). These processes take place in the outer-membrane-bounded periplasmic space and may involve more complex regulatory mechanisms rather than the simple blocking mechanism suggested by our two repression rules.

On the other hand, both the informative 4-mer motifs TTTT and CATT appeared once on the CRP-binding site of *exuT*. Similarly, *exuT* is also annotated with membrane-related GO terms (GO:0055085 - transmembrane transport, GO:0015736 - hexuronate transport, and GO:0005886 - plasma membrane). We assume that *exuT* may involve more complex activation mechanisms rather than Class I and II mechanisms.

The PredCRP method can be applied to predict the regulatory roles of other global regulators in *E. coli*. First, one can download the dataset of TF-binding sites from RegulonDB, and use the feature extraction programme from https://github.com/NctuICLab/PredCRP. This programme can retrieve the TFs of interest with a given evidence level (strong or weak). Consequently, one can use a similar process like that described in the Materials and Methods section to train a predictor of regulatory roles.

## Materials and Methods

Figure 1 illustrates the main components of PredCRP, consisting of an accurate model (PredCRP-model) and a set of interpretable rules (PredCRP-ruleset), including (1) establishment of datasets of CRP-binding sites, (2) feature extraction from CRP-binding sites, (3) feature selection in cooperation with an SVM, (4) PredCRP-ruleset based on the decision tree method C4.5, (5) an accurate PredCRP-model and (6) experimental validation of the regulatory roles of CRP. To provide the CRP prediction service to the scientific community, PredCRP-model can be accessed and downloaded at https://github.com/NctuICLab/PredCRP.

### Datasets of CRP-binding sites

A dataset (named CRPS) consisting of 169 CRP-binding sites with regulatory roles supported by strong evidence was established to train and evaluate this work. The CRPS dataset was retrieved from the RegulonDB database (version 8.8)^29^, containing up-to-date information on *E. coli*. The retrieval procedure of CRPS was given as follows:

Step 1:  Retrieve TF-binding sites where the “TF name” column was annotated with ‘CRP’ from the RegulonDB database.

Step 2:  Categorise CRP-binding sites into strong and weak evidence groups using the “evidence” property obtained from the RegulonDB database.

Step 3:  Remove redundant CRP-binding sites with the same transcription start site by checking the leftmost and rightmost positions in the genome.

Step 4:  Filter out the CRP-binding sites with lengths not equal to 22 base pairs.

The strong-evidence group in the RegulonDB v8.8 database is the set of sites selected by performing mutation experiments on TF-binding sites^29^. As a result, the CRPS dataset consisting of 169 CRP-binding sites was randomly divided into training and test datasets in a ratio of 2:1, referred to as CRPS-TRN and CRPS-TST, respectively. CRPS-TRN consists of 24 repression sites and 89 activation sites, while CRPS-TST consists of 12 repression sites and 44 activation sites. The CRPS dataset was subsequently used to design and evaluate this work.

An additional dataset (CRPS-TST-2) was used as an independent test for evaluating the PredCRP-model. The CRPS-TST-2 dataset consists of the strong evidence CRP-binding sites that appeared in version 9.4 but not version 8.8 of the RegulonDB database. CRPS-TST-2 contains 12 sites of activation and 8 sites of repression.

To explore the regulatory roles of CRP on all CRP-regulated genes, we enumerated all possible CRP-binding sites and screened putative ones using interpretable rules. The possible CRP-binding sites were first retrieved from the RegulonDB database with weak-evidence annotation (CRPW). The weak evidence was annotated using the following methods^29^: (1) binding in cellular extracts, (2) gene expression analysis, (3) similarity to consensus sequences, (4) reaction blocked in a mutant, (5) inferred by a curator, (6) inferred from a mutant phenotype, and (7) prediction results. The conserved sequence of CRP-binding sites, -NNNTG_5_TG_7_ANNNNNNTC_16_AC_18_ANNN-, a well-known palindromic sequence, is bound by CRP^25–27^. In this consensus sequence, the nucleic acid G at positions 5 and 7 and the nucleic acid C at positions 16 and 18 play crucial roles in providing binding free energy between CRP and the CRP-binding site^30–32^. The detailed CRP-binding ability in these four positions is provided in Supporting Information. The consensus sequence criterion can help to determine whether a site is a binding site or not but cannot determine the regulatory roles of CRP directly.

We selected only the four important sites in which the nucleic acids at positions 5, 7, 16 and 18 were G, G, C, and C, respectively. Consequently, 23 previously unobserved, putative CRP-binding sites with regulatory roles predicted by PredCRP-model and PredCRP-ruleset were identified. Although the 23 putative CRP-binding sites have high probability to be CRP-binding sites according to the consensus sequence criterion, we further proved the 23 sites as CRP-binding sites by referring to the EcoCyc database (see Supporting Information). The 23 predicted CRP-regulated interactions are given in Figure S6, which were validated by qPCR experiments.

### Feature extraction from CRP-binding sites

A comprehensive feature set of CRP-binding sites was established, comprising 380 features, including 17 features from a location-dependent descriptor (Table S3)^18^, three features from a physicochemical property descriptor, 320 features from the composition descriptors (256 features of 4-mer motif composition descriptor and 64 features of the 3-mer motif composition descriptor), and 40 features from a global sequence descriptor. This work extended the length of CRP-binding sites from 22 to 42 base pairs by respectively adding *k* base pairs of flanking nucleotides to the regions upstream and downstream of a CRP-binding site for considering interactions within a *cis*-regulatory region^18^. In this work, *k* = 10 was used. The detailed feature extraction is given in Supplementary Information.

### Feature selection in cooperation with a support vector machine

Selecting a minimal set of *m* informative features from *n* = 380 features while maximising the prediction accuracy of the SVM classifier using these *m* features is a bi-objective combinatorial optimisation problem C(*n*, *m*). To deal with this large parameter optimisation problem, the inheritable bi-objective combinatorial genetic algorithm (IBCGA) was used^28^. The IBCGA, using an intelligent evolutionary algorithm^33^, can simultaneously obtain a set of solutions, *S*_*r*_, where *r* = *r*_*start*_, *r*_*start*_ + 1, …, *r*_*end*_ in a single run using an inheritance mechanism to efficiently search for the solution *S*_*r*+1_ to C(*n*, *r* + 1) by inheriting a good solution *S*_*r*_ to C(*n*, *r*). *S*_*m*_ is the best solution among solutions *S*_*r*_. The IBCGA can efficiently solve large-scale feature selection problems and is useful for deriving an optimised SVM model^34,35^. The intelligent evolutionary algorithm is good at solving large parameter optimisation problems, such as inferring roles within a large-scale quantitative gene regulatory work^36^.

The optimised SVM classifier with the *m* selected informative features is evaluated using the following measurements: prediction accuracy (ACC), sensitivity (SEN), specificity, and Matthews’s correlation coefficient^37^. To provide prediction service to the scientific community, we developed a user-friendly tool based on the SVM model. The detailed algorithm is shown in Supporting Information. The parameter settings of the feature selection module are shown in Table S4.

### PredCRP-ruleset based on C4.5

The *m* informative features for predicting the regulatory roles of CRP and CRPS were utilised to develop a rule acquisition method based on the decision tree method C4.5^38^. A set of if-then rules for distinguishing activators from repressors can be derived from the established decision tree. The interpretable if-then rules are used to further elucidate the regulatory roles of CRP.

### An accurate model PredCRP-model

In this work, the fitness function of the IBCGA is the prediction accuracy of *k*-fold cross-validation. The numbers of repressors and activators for training the SVM model are 24 and 89, respectively. To maximise the number of training repressors used, *k* = 24 was used in this study. The input of the IBCGA is *n*-dimensional feature vectors of CRP-binding sites with regulatory roles in CRPS-TRN, and the output contains a set of *m* selected features and an SVM-based classifier with associated parameter settings of *γ* and *C*. PredCRP-model is the optimised SVM classifier with the *m* selected informative features. PredCRP-model is evaluated using the following measurements: prediction accuracy (ACC), sensitivity (SEN), specificity, and Matthews’s correlation coefficient^37^.

### Experimental validation of the regulatory roles of CRP

#### Experimental design of quantitative PCR experiments

CRP is a well-known TF governing carbohydrate metabolism^39^. When *E. coli* grows in a medium containing glucose, the intracellular cAMP concentration is low^40^. The DNA-binding ability of CRP is regulated by the cAMP concentration^39,41^. A quantitative measurement of CRP-mediated transcriptional regulation was performed by measuring the gene expression level in an *E. coli* K12 strain lacking the *cyaA* gene producing endogenous cAMP^42^, under various cAMP concentrations. Mutant strains used were *E. coli* BW25113 derivatives generated from the Keio collection system and provided by the National Institute of Genetics of Japan^43^. The regulatory roles of CRP on a CRP-regulated gene can be determined using the difference between the expression levels of the wild type with the cAMP concentrations of 0 and 1 mM.

#### Quantitative measurement of gene expression levels

The quantitative PCR method can obtain expression profiles of genes of interest in a high-throughput and accurate manner^22^. In this work, the same experimental conditions were used^44^. RNA isolation was based on the suggested protocol in the TRI Reagent-RNA Kit (Molecular Research Center, Cincinnati, OH, USA). RNA samples for quantitative PCR were pre-treated with DNase I (Promega, Madison, WI, USA). The DNA primers used in the quantitative PCR were designed by Primer Express software (Applied Biosystems, Foster City, CA, USA) and their complements (Table S5). DNA was synthesised using SuperScript III Reverse Transcriptase (Invitrogen, Carlsbad, CA, USA). The quantitative PCR experiments were conducted in an ABI PRISM® 7000 instrument (Applied Biosystems, Foster City, CA, USA) using the SYBR Premix Ex Taq reagent (Takara, Tokyo, Japan). All quantitative PCR experiments were performed with two replicates.

## Electronic supplementary material

Supporting information
