# Supplementary material for: PredCRP: predicting and analysing the regulatory roles of CRP from its binding sites in Escherichia coli
Source: Sci Rep. 2018 Jan 17;8:951. doi: 10.1038/s41598-017-18648-5 (PMC5772556; doi:10.1038/s41598-017-18648-5)
Supplement: Supplementary file 1 — Supporting information [file 41598_2017_18648_MOESM1_ESM.pdf]

Supporting information

PredCRP: predicting and analysing the regulatory roles of CRP from its binding sites in *Escherichia coli*

Ming-Ju Tsai[1], Jyun-Rong Wang[1], Chi-Dung Yang[2,3], Kuo-Ching Kao[1], Wen-Lin Huang[4], Hsi-Yuan Huang[5], Ching-Ping Tseng[2], Hsien-Da Huang[1,2], and Shinn-Ying Ho[1,2*]

[1]Institute of Bioinformatics and Systems Biology, National Chiao Tung University, Hsinchu, Taiwan

[2]Department of Biological Science and Technology, National Chiao Tung University, Hsinchu, Taiwan

[3]Institute of Population Health Sciences, National Health Research Institutes, Miaoli, Taiwan

[4]Department and Institute of Industrial Engineering and Management, Minghsin University of Science and Technology, Hsinchu, Taiwan

[5]Department of Laboratory Medicine, China Medical University Hospital, Taichung, Taiwan

*Corresponding author

Email address:

SYH: syho@mail.nctu.edu.tw

Materials and Methods

Datasets of CRP-binding sites

A survey of the 23 putative CRP-binding sites

The DNA sequence, -NNNTG$_5$TG$_7$ANNNNNNNTC$_{16}$AC$_{18}$ANNN-, a well-known palindromic sequence, is bound by CRP [1-3]. (a G to C mutation at position 5 and a C to G mutation at position 16) reduced CRP-binding ability significantly and used as the negative control in normal EMSA experiments [4,5]. Careful observation of all of the 23 putative CRP-binding sites on promoter regions which we used in this study reveals that all DNA sequences four matched base on the G$_{5,}$ G$_{7,}$ C$_{16}$ and C$_{18}$ at the palindromic sequence, except the CRP consensus sequence on the *ycdZ* promoter, which have one mutation on C$_{18}$ change to T$_{18}$. Based on the above reasons, the experiment results of EMSA and the related references from the EcoCyc database [6] listed in Table S1, we can conclude and generalize that 23 putative CRP-binding sites are CRP-binding sites.

Feature extraction of the CRP-binding sites

Composition descriptor

The composition of 4-mer motifs was calculated based on the number of all 4-mer motifs (i.e. from AAAA to TTTT), which may be related to the codon usage of polymerase [7]. The number of all 4-mer motifs were calculated using the following formula.

$$N_{4-mer} = (\frac{N_{AAAA}}{L}, \frac{N_{AAAC}}{L}, \dots, \frac{N_{TTTT}}{L}) \qquad (1)$$

In equation (1), L is the length of the binding site sequence and the N_{AAAA} to N_{TTTT} are defined as the occurrence frequency of a specific 4-mer in a given binding site sequence. Similarly, The composition of 3-mer was calculated using the following formula.

$$N_{3-mer} = (\frac{N_{AAA}}{L}, \frac{N_{AAC}}{L}, \dots, \frac{N_{TTT}}{L}) \qquad (2)$$

Location-dependent descriptor

Although the regulatory roles of CRP have been previously examined [8], few quantitative studies have been conducted. For instance, the distribution of transcription factor (TF)-binding site locations for activators and repressors has been examined in studies characterising the roles of regulation of TFs in *E. coli*. However, the accuracy of predicting each TF using the distribution of TF binding site locations has not been reported. In this work, the 17 features from the location-dependent location descriptor were integrated to predict the regulation mode of CRP in *E. coli*. The novel location descriptor include location-dependent knowledge such as the operator centre position of the CRP-binding site, the size of the overlap regions between a CRP-binding site to the regions of specific mechanisms (Table S3). However, these sequence descriptors have not been reported previously for predicting the regulation roles of TFs in *E. coli*.

Physicochemical property descriptor

For a given DNA sequence, the 3 DNA physicochemical properties represent the average absorption maxima, molecular weight, and molar absorption coefficient of the given sequence, respectively [9].

Global sequence descriptor

The global sequence descriptior of promoter/non-promoter sequences contains four parts, entropy density profile (EDP), single nucleotide composition, transition, and DNA nucleotide distribution [10]. The EDP model is a globally statistical descriptor of DNA sequences based on Shannon's artificial linguistic description for a DNA sequence of finite length [11]. Six EDP descriptors, including EDP_{EQ}, EDP_{EH}, and EDP_{Ei} (EDP_{EA}, EDP_{EC}, EDP_{EG}, and EDP_{ET}) are defined as follows:

$$EDP_{EQ} = q_A^2 + q_C^2 + q_G^2 + q_T^2 \quad (3)$$

$$\mathrm{EDP_{EH}} = -\sum_{\mathrm{i}} \mathrm{q_i} \log \mathrm{q_i} \quad (4)$$

$$\mathrm{EDP_{E_i}} = \frac{-1}{\mathrm{EDP_{EH}}} \mathrm{q_i} \log \mathrm{q_i} \quad (5)$$

In these formulas, $\mathrm{EDP_{EQ}}$ and $\mathrm{EDP_{Ei}}$ are statistical quantities, $\mathrm{q_i}$ is the single nucleotide composition, *i* is the index that specifies the nucleotides (A, C, G, and T), and $\mathrm{EDP_{EH}}$ is Shannon's entropy. Additionally, the $\mathrm{q_i}$ of four nucleotides (A, C, G, and T) is also included in the global sequence descriptor.

Features of the transition descriptor, T(α, β), are used to characterise the percent frequency with which α is followed by β or β is followed by α, where α is not equal to β. The six transition frequencies include T(A, C), T(A, G), T(A, T), T(C, G), T(C, T), and T(G, T). For example, for the S20 sequence, there are four transitions of type T(A, C), CATAGCCATTGCATGACCCG; the letters shown in bold indicate that the value of CRP T(A, C) is 21.05 %(4/19).

The final part of the global sequence descriptor is the locations of a certain nucleotide (A, C, G, and T) in n-th segments of a given sequence divided by the length of the given sequence. In this work, n = 5 was used. For example, the S20 sequence, CATAGCCATTGCATGACCCG, The nucletiode "A" is shown at the positions 2, 4, 8, 13, and 16. Hence, the features of first A, second A, third A, fourth A and fifth A have values of 10% (2/20), 20% (4/20), 40% (8/20), 65% (13/20), and 80% (16/20), respectively.

Feature selection in cooperation with an SVM

Step 1: Each sample is represented as an *n*-dimensional feature vector $p = [p_1, p_2, \ldots, p_n]$ In this work, n = 380 was used.

Step 2: Each IBCGA-chromosome consists of binary genes f_i from which to select 380 features and two 4-bit genes for encoding the kernel parameter γand the cost parameter (*C*). The corresponding feature p_i (the i-th feature) is excluded from the SVM classifier if $f_i = 0$, and p_i is included if $f_i = 1$. Let m be the sum of f_i. The $\gamma > 0$ determines how the samples are transformed into a

high-dimensional search space. The cost parameter C > 0 of the SVM classifier adjusts the penalty of total error. These two parameters C and γ must be tuned to obtain the best prediction performance. In this work, $\gamma \in \{2^{-8}, 2^{-7}, \ldots, 2^{7}\}$and $C \in \{2^{-8}, 2^{-7}, \ldots, 2^{7}\}$

Step 3: The fitness function is the prediction accuracy of k-fold cross-validation using the LIBSVM classifier [12] with the *m* selected informative features and the SVM parameters (γ and *C*) by decoding the IBCGA-chromosome. In this study, a popular kernel function which is a radial basis function $\exp(-\gamma\,||x^i - x^j||^2)$ was adopted. x^i and x^j were training samples and γ was a kernel parameter. The parameter settings of IBCGA are shown in Table S4. In this study, k = 24 was used.

Step 4: All solutions for S_r from $r = r_{start}$ to r_{end} are obtained using IBCGA. Let S_m be the most accurate solution with *m* selected features among all solutions from $C(n, r_{start})$ to $C(n, r_{end})$ search space. In this study, r_{start} = 10 and r_{end} = 40 were used.

Step 5: IBCGA use mechanisms of randomisation and are therefore characterised as non-deterministic because the results of individual runs are not always the same. Therefore, Steps 3 and 4 are performed for *R* independent runs to obtain the best *R* number of discrete runs to obtain the best *R* solutions. In this study, *R* = 30 was used.

Inference process of interpretable rules

Let *l* and *r* be the left end (the start position of CRP-binding site) and the right end (the end position of CRP-binding site) of a given CRP-binding site. Since the length of a CRP-binding site is 22, there exists the following equations. The nodes of the decision tree are numbering in both top-to-down and left-to-right manners (Figure S8).

$$l + 21 = r \tag{6}$$

$$r - 21 = l \tag{7}$$

Activation rule 1

Step1: IF (Node1 is equal to 0) THEN (r < -10 OR l > 2)

Step2:SINCE (r < -10) THEN (l + 21 < -10)

Step3: SINCE (l + 21 < -10) THEN (l < -31)

Step4:HENCE, IF (Node1 is equal to 0) THEN (*l* < -31 OR *l* > 2)

Step5: IF (Node 3 <= 11.5) THEN ((*r* - 10.5 <= -60) OR (*l* + 10.5 >= 60))

Step6: SINCE (*r* - 10.5 <= -60) THEN (*l* + 21 <= -49.5)

Step7: SINCE (*l* + 21 <= -49.5) THEN (*l* <= -70.5)

Step8: HENCE, IF (Node 3 <= 11.5) THEN (*l* <= -70.5 OR *l* >= 49.5)

Step9: IF ((Node1 is equal to 0) AND (Node 3 <=11.5) AND (the TTTT composition <=2)) THEN (a given CRP-binding site tends to be an activator)

Activation rule 1 states that if a given CRP-binding site statisfy (*l* < = -70.5 OR *l* >= 49.5) AND (the TTTT composition <=2)) then it tends to be an activator. *l* < = -70.5 is equivalent to *r* <= -49.5. The inference process of the location criteria is shown in Figure S2.

Activation rule 2

Step1: IF (Node1 is equaul to 0) THEN (*r* < -10 OR *l* > 2)

Step2: SINCE (*r* < -10) THEN (*l* + 21 < -10)

Step3: HENCE, IF (Node1 is equal to 0) THEN (*l* < -31 OR *l* > 2)

Step4: IF (Node3 > 11.5) THEN ((*r* - 10.5 > -60) OR (*l* + 10.5 < 60))

Step5: SINCE (*r* - 10.5 > -60) THEN (*r* > -49.5)

Step6: SINCE (*r* > -49.5) THEN (*l + 21* > -49.5)

Step7: SINCE (*l + 21* > -49.5) THEN (*l* > -70.5)

Step8: HENCE, IF (Node3 > 11.5) THEN (*l* > -70.5 OR *l* < 49.5)

Step9: IF (Node5 > 15.5) THEN ((*r* - 14.5 > -95) AND (*l* + 14.5 < -35))

Step10: SINCE (*r* - 14.5 > -95) THEN (*l* + 21 > -80.5)

Step11: SINCE (*l* + 21 > -80.5) THEN (*l* > -101.5)

Step12: HENCE, IF (Node5 > 15.5) THEN ($-101.5 < l < -49.5$)

Step13: IF ((Node1 is equal to 0) AND (Node3 > 11.5) AND (Node5 > 15.5)) THEN ($-70.5 < l < -49.5$)

Step14: SINCE ($l < -49.5$) THEN ($r - 21 < -49.5$)

Step15: SINCE ($r - 21 < -49.5$) THEN ($r < -28.5$)

Step16: IF ((Node1 is equal to 0) AND (Node3 > 11.5) AND (Node5 > 15.5) AND (the AACG composition is equal to 0)) THEN (a given CRP-binding site tends to be an activator)

Activation rule 2 states that if a given CRP-binding site statisfy (($-70.5 < l < -49.5$) AND (the AACG composition is equal to 0)) then it tends to be an activator. ($-70.5 < l < -49.5$) is equivalent to $-70.5 < \mathit{region} < -28.5$. The inference process of the location criteria is shown in Figure S3.

Repression rule 1

Step1: IF (Node1 is equal to 0) THEN ($r < -10$ OR $l > 2$)

Step2: SINCE ($r < -10$) THEN ($l + 21 < -10$)

Step3: SINCE ($l + 21 < -10$) THEN ($l < -31$)

Step4: HENCE, IF (Node1 is equal to 0) THEN ($l < -31$ OR $l > 2$)

Step5: IF (Node 3 > 11.5) THEN (($r - 10.5 > -60$) OR ($l + 10.5 < 60$))

Step6: SINCE ($r - 10.5 > -60$) THEN ($r > -49.5$)

Step7: SINCE ($r > -49.5$) THEN ($l + 21 > -49.5$)

Step8: SINCE ($l + 21 > -49.5$) THEN ($l > -70.5$)

Step9: HENCE, IF (Node 3 > 11.5) THEN (($l > -70.5$) OR ($l < 49.5$))

Step10: IF (Node5 <= 15.5) THEN (($r - 14.5 <= -95$) OR ($l + 14.5 >= -35$))

Step11: SINCE ($r - 14.5 <= -95$) THEN ($r <= -80.5$)

Step12: SINCE ($r <= -80.5$) THEN ($l + 21 <= -80.5$)

Step13: HENCE, IF (Node5 <= 15.5) THEN(*l* <= -101.5 OR *l* >= -49.5)

Step10: IF ((Node1 is equal to 0) AND (Node 3 > 11.5) AND (Node5 <= 15.5) AND (the TTAC composition is equal to 0))

THEN (a given CRP-binding site tends to be a repressor)

Repression rule 1 states that if a given CRP-binding site statisfy ((-49.5 <= *l* < -31) OR (2 < *l* < 49.5))AND (the TTAC composition is equal to 0)) then it tends to be a repressor. (-49.5 <= *l* < -31) is equivalent to -49.5 <= *region* < -10. 2 < *l* < 49.5 is equivalent to

2 < *region* < 70.5. The inference process of the location criteria is shown in Figure S4.

Repression rule 2

Step1: IF (Node1 is equal to 0) THEN ((*r* > -10) OR (*l* < 2))

Step2: SINCE (*r* > -10) THEN (*l* + 21 > -10)

Step3: SINCE (Step1 AND Step2) THEN (-31 < *l* < 2)

Step4: SINCE (*l* < 2) THEN (*r* – 21 < 2)

Step5: SINCE (*r* – 21 < 2) THEN (*r* < 23)

Step6: IF (Node1 is equal to 0 AND (the GAGC composition is equal to 0) AND (the TTAC composition is equal to 0)) THEN (a given CRP binding site tends to be a repressor)

Repression rule 2 states that if a given CRP-binding site statisfy ((-31 < *l* < 2) AND (the GAGC composition is equal to 0) AND (the TTAC composition is equal to 0)) then it tends to be a repressor. (-31 < *l* < 2) is equiavalent to -31 < *region* < 23. The inference process of the location criteria is shown in Figure S5.

Inference of relative quantity in real-time qPCR

In this study, To determine the regulatory roles of the studied sequence, a relative method can be used, where *16S* rRNA gene is a calibrator. First, internal control for each gene, difference between ΔCt of studied gene and control gene (*16S*) is calculated, then subtract between (so the value of the "ΔΔCt") ΔCt of sample with 1mM and ΔCt of the calibrator (0mM). Normalized value of the expression level relative to the calibrator is determined by the formula [13]: Relative quantity = $2^{-\Delta\Delta Ct}$. The results of qPCR experiment are shown in Table S2.

Supplementary Tables

Table S1. Analysis of the crucial binding positions of the 23 putative CRP-binding sites

10bp + CRP binding sites + 10bp	CRP-regulated gene	Reference
gttatctataTTAT**G**T**G**ATCTAAAT**C**A**C**TTTTaagtcagagt	*aaeR*	14
caaaggcaaaAAAT**G**T**G**ATTTCGTA**C**A**C**ATCTgatttcactg	*ldtB*	15
cagtgaaatcAGAT**G**T**G**TACGAAAT**C**A**C**ATTTtttgcctttg	*ybiT*	14,15
ttgttgcataAAAT**G**T**G**TGCTCGAT**C**T**C**ATTCatggccgcgt	*ycdZ*	16
aacaattttcTGAC**G**T**G**ATCTTCAT**C**A**C**AAATaatgacagtt	*idnDOTR*	17
actctgacttAAAA**G**T**G**ATTTAGAT**C**A**C**ATAAtatagataac	*aaeXAB*	14
gtaatcccaaAGCG**G**T**G**ATCTATTT**C**A**C**AAATtaataattaa	*aspA-dcuA*	18,19
cccgaaacaaAAAT**G**T**G**ATACCAAT**C**A**C**AGAAtacagcttat	*caiTABCDE*	20-22
atattcccacATTT**G**T**G**ATGGCTCT**C**A**C**CTTTtaaagttgta	*exuT*	23
gcgattacacTGAT**G**T**G**ATTTGCTT**C**A**C**ATCTttttacgtcg	*galP*	24
caatctccgcGAGC**G**T**G**CCAGTTTT**C**A**C**ATTCttcagttgca	*grpE*	15
atacctcactTCTC**G**T**G**ATCAAGAT**C**A**C**ATTCtcgctttccc	*hyfABCDEFGHIJR-focB*	25
tttttcacaaATTT**G**A**G**AGTTGAAT**C**T**C**AAATcatatcaaaa	*malS*	26,27
aaagcccgaaAAAT**G**T**G**CTGTTAAT**C**A**C**ATGCctaagtaaaa	*mlc*	28
gacgtcattaTAGT**G**T**G**TGTCAGAT**C**T**C**GTTTtccttaacca	*nupC*	15,29,30
aaccgcagctATTT**G**T**G**AATCTTTT**C**A**C**AGTTtaaattcccc	*preTA*	31
aaaatgcccgAGAT**G**T**G**AAGCAAAT**C**A**C**CCACttaatgccgt	*rhaT*	15,32
taaatgttgtTATC**G**T**G**ACCTGGAT**C**A**C**TGTTcaggataaaa	*sdhCDAB-sucABCD*	15,33
actggtcgtaTGCG**G**T**G**ACGGAGTT**C**A**C**CCTTtacgcctcct	*sfsA-dksA*	34
acggcattaaGTGG**G**T**G**ATTTGCTT**C**A**C**ATCTcgggcatttt	*sodA*	15
ttttaaagatTAAT**G**C**G**ATCTATAT**C**A**C**GCTGtgggtattgc	*uidABC*	35
ttccattttaTTTT**G**C**G**AGCGAGCG**C**A**C**ACTTgtgaattatc	*xylAB*	36
gataattcacAAGT**G**T**G**CGCTCGCT**C**G**C**AAAAtaaaatggaa	*xylFGHR*	36

The G_5, G_7, C_{16} and C_{18} are highlighted in bold.

Table S2. The results of real-time qPCR experiments.

Gene	0mM		1mM		0mM (ΔCt)		1mM (ΔCt)		ΔΔCt		RQ*		AVG RQ	Roles*
	R1*	R2*	R1	R2	R1	R2	R1	R2	R1	R2	R1	R2		
16s	10.3	10.6	10.2	10.2	-	-	-	-	-	-	-	-	-	-
aaeR	30.4	29.6	32.3	31.7	20.1	19.0	22.1	21.5	2.1	2.6	0.2	0.2	0.2	R
aaeX	27.4	28.0	25.4	26.2	17.1	17.4	15.2	16.0	-2.0	-1.4	3.9	2.7	3.3	A
aspA	25.6	24.2	22.4	22.4	15.3	13.6	12.2	12.2	-3.2	-1.4	8.9	2.6	5.8	A
caiT	29.0	28.6	25.6	25.4	18.7	18.0	15.4	15.2	-3.3	-2.7	10.1	6.6	8.4	A
exuT	24.5	24.5	21.2	21.0	14.2	13.9	11.0	10.8	-3.3	-3.1	9.6	8.8	9.2	A
galP	27.5	29.0	25.5	25.7	17.2	18.3	15.3	15.5	-1.9	-2.8	3.7	7.2	5.4	A
grpE	23.4	23.6	22.1	21.5	13.1	12.9	11.9	11.3	-1.2	-1.6	2.3	3.1	2.7	A
hyfA	30.0	29.9	27.4	27.6	19.7	19.3	17.2	17.4	-2.5	-1.9	5.5	3.8	4.7	A
idnD	20.5	20.6	18.2	18.6	10.3	10.0	7.9	8.4	-2.3	-1.6	5.0	3.0	4.0	A
ldtB	26.9	26.5	29.1	29.2	16.6	15.9	18.9	19.0	2.3	3.1	0.2	0.1	0.2	R
malS	29.9	30.5	27.0	26.0	19.6	19.9	16.8	15.9	-2.8	-4.0	6.9	16.3	11.6	A
mlc	32.4	31.9	27.0	27.3	22.2	21.2	16.8	17.1	-5.4	-4.2	41.9	18.0	30.0	A
nupC	25.9	25.1	23.5	23.4	15.6	14.4	13.3	13.2	-2.3	-1.2	4.8	2.4	3.6	A
preT	20.8	19.5	17.9	17.9	10.5	8.9	7.7	7.7	-2.8	-1.2	7.1	2.3	4.7	A
rhaT	24.3	24.1	21.0	23.0	14.0	13.5	10.8	12.8	-3.2	-0.7	9.3	1.6	5.5	A
sdhC	21.3	19.9	16.6	15.8	11.0	9.2	6.4	5.6	-4.6	-3.7	24.6	12.7	18.7	A
sfsA	18.6	18.2	17.4	17.2	8.3	7.6	7.2	7.0	-1.1	-0.6	2.2	1.5	1.8	A
sodA	17.3	16.0	17.2	15.1	7.0	5.4	7.0	4.9	0.1	-0.5	1.0	1.4	1.2	A
uidA	22.0	23.8	19.6	21.6	11.7	13.2	9.4	11.4	-2.3	-1.8	5.0	3.5	4.3	A
xylA	24.0	24.1	22.7	21.3	13.7	13.5	12.4	11.1	-1.2	-2.5	2.4	5.5	3.9	A
xylF	23.6	21.5	19.3	18.6	13.3	10.9	9.0	8.4	-4.2	-2.5	18.8	5.7	12.2	A
ybiT	20.1	20.4	23.2	23.6	9.8	9.7	12.9	13.4	3.2	3.6	0.1	0.1	0.1	R
ycdZ	19.1	19.2	24.1	24.2	8.8	8.5	13.9	14.1	5.0	5.5	0.0	0.0	0.0	R

Roles, A stands for activation, and R stands for repression; RQ: Relative quantity; R1: Replicate 1; R2: Replicate 2

Table S3. The location-dependent descriptors from literature review

**Index**	**Location Description**
L1	The CRP-binding site located on the regions of upstream or downstream to the transcription start site.
L2	Distance from the centre position of the CRP-binding site to the transcription start site
L3	The size of the overlap region between the CRP-binding site and the region from -35 to -10.
L4	Distance between the CRP-binding site to the region from -35 to -10.
L5	The CRP binding site located on upstream or downstream to the region from -35 to -10. The upstream is defined as the region lower than -35 and the downstream is defined as the region larger than -10
L6	The size of the overlap region between the CRP-binding site and the region from -10 to 2. This

	feature may involve in the transcription bubble mechanism.
L7	The size of the overlap region between the CRP-binding site and other repressor binding sites
L8	The size of the overlap region between the CRP-binding site and other activator binding sites
L9	The CRP binding site located on forward strand or reverse strand
L10	The size of the overlap region between the CRP-binding site and the region from -95 to -60. This feature is consistent with the Class I rule.
L11	Thes size of the overlap region between the CRP-binding site and the region from -50 to 35. This feature is consistent with the Class II rule.
L12	The size of the overlap region between the CRP-binding site and the region from -60 to 60. This feature may involve in the three mechanisms, 1) activation by DNA conformation change, 2) repression by DNA looping and 3) cooperative repression
L13	The size of overlap region between the CRP-binding site and the region from position -10 to 60. This feature may involve in the repression by roadblock mechanism.
L14	The size of the overlap region between the CRP-binding site and the region from -95 to -10. This feature may involve the repression by activator modulation mechanism
L15	The size of the overlap region between the CRP-binding site and the region from -95 to -35. This feature may involve in the cooperative activation mechanism.
L16	The size of the overlap region between the CRP-binding site and the region from -10 to 10. This feature may involve in the promoter escape regulation mechanism.
L17	The number of escaped promoters.

Transcription start site denotes +1;

Table S4. The used control parameters of IBCGA

**Parameter**	**Value**
Population size N_{pop}	50
Selection probability p_s	0.2
Crossover probability p_c	0.8
Mutation probability p_m	0.05
Factor number of orthogonal arrays	7
Maximum generations G_{max}	60

Table S5. The used DNA primers for the quantitative PCR experiments

Gene	Reverse Sequence	Forward sequence
aaeR	CAGCTCCCCACGATTGATCT	GACTCGCCTGATCCCACAAG
aaeX	AGCAATAGAGCGCGGTGTTG	GTGGTGTTTGGGCTGTCCTT
aspA	TTAGCAGTGATGCCGTTAATGC	CACTGTTACCATGGCAGCAGAA
caiT	AACACCCAACCCAGCATCAG	CCTGCAACTGGACGCTATCA
hyfA	ACATATTGCGCATGGGCATT	GATGCGATCCAACTCAACGA
idnD	ATGGGTCCAACACCGGAAAT	ATGGCTTTTGCCGAACCTTT
ldtB	TTGGCACAATTTCCTGACCTT	GACGGCAGCCGTTATATTGAAG
malS	CGTTCACCCAGCGATTTTTT	ATGAACCACACCGGCTATGC
mlc	CCTGCAACAGACGAATCAACA	GTCAGCACATCAGCGTTGAGA
nupC	CATGTATGCACCAACGATGGA	CGGCAAAATCTCCCGTAATC
preT	AATTTTATCTGCCGCCATCGT	TCGATGGATTCACGCCAGTA
rhaT	ACAGTGGATCGACGCCAAGT	GGTGATGTGCGGCATTTTCT
sdhC	GATTTTGGCGGAGCGTTTAC	AGGTATTCGCCACATGATGATG
sfsA	CTACCCCCCTCTGTTGAGCTT	CAGCGTGCGGTTATCTTTTTC
sodA	GCTGCTTCGTCCCAGTTCAC	CGATTATGGGCCTGGATGTG
uidA	GCACCATCAGCACGTTATCG	GCAGTGAAGGGCGAACAGTT
xylA	GATGCACCGGAGACAAATGA	TGAAGATGGCGAGCTGGATAA
xylF	GTGTTTCTTCATTGCCATTTGC	CTGCACACGCCAAAGAAGTC
ybiT	CCGCCGAGGATCTTCATAAA	GTTCGGCAGTAAGCCGTTGT
ycdZ	GCAGCTGTTTGGCCTGAATAC	CCACATCTGGAAATTCTCGGTTAT
tnaA	GGGTTCTGCACTCGGTGTACA	CCGCGAAACCTACAAATATGC
exuT	TGCATTACGATTGCCCATACC	AAAGCCAGCTCCGAATGGTT
grpE	GTTACCTGGCGCAACGTCAT	TGCGTAAGTTTGGCGTTGAA

Supplementary Figures

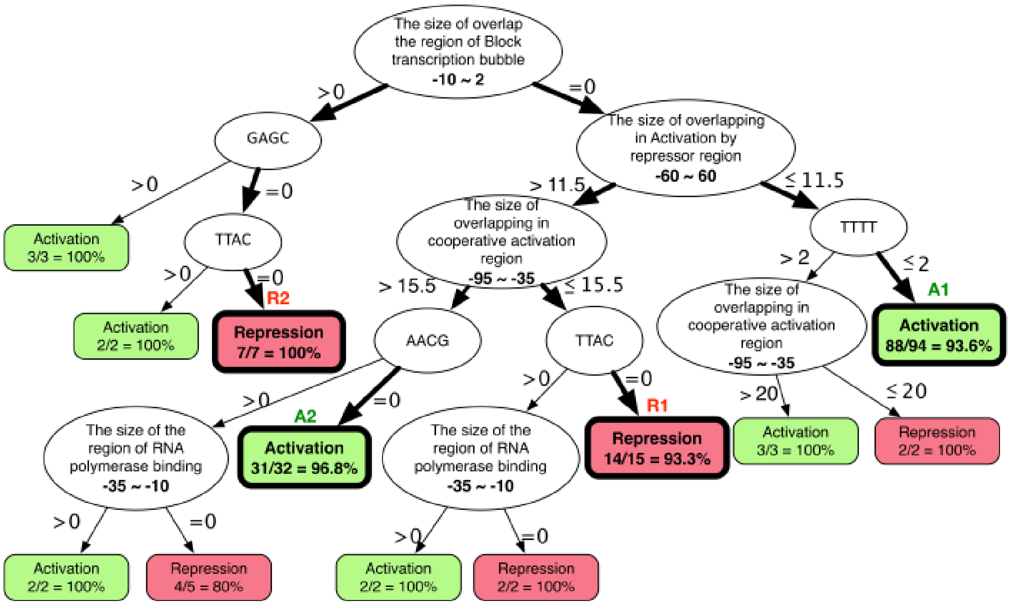

Figure S1. The decision tree was established using 12 informative features.

This decision tree is pruned using the confidence level 25%. The four prediction rules with a high cover rate of CRPS were selected. These corresponding paths are highlighted with thick lines.

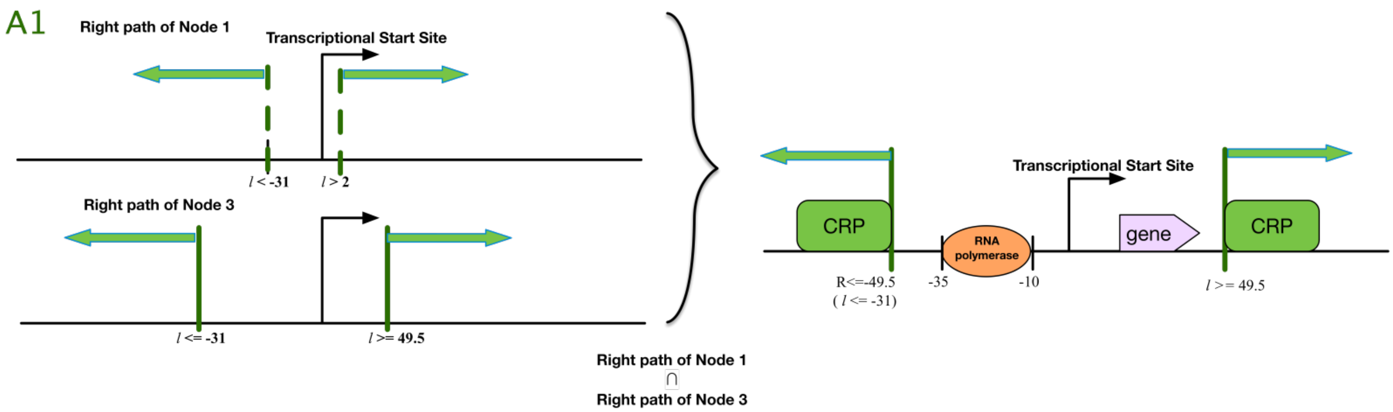

Figure S2. The inference process of the location critieria of activation rule 1.

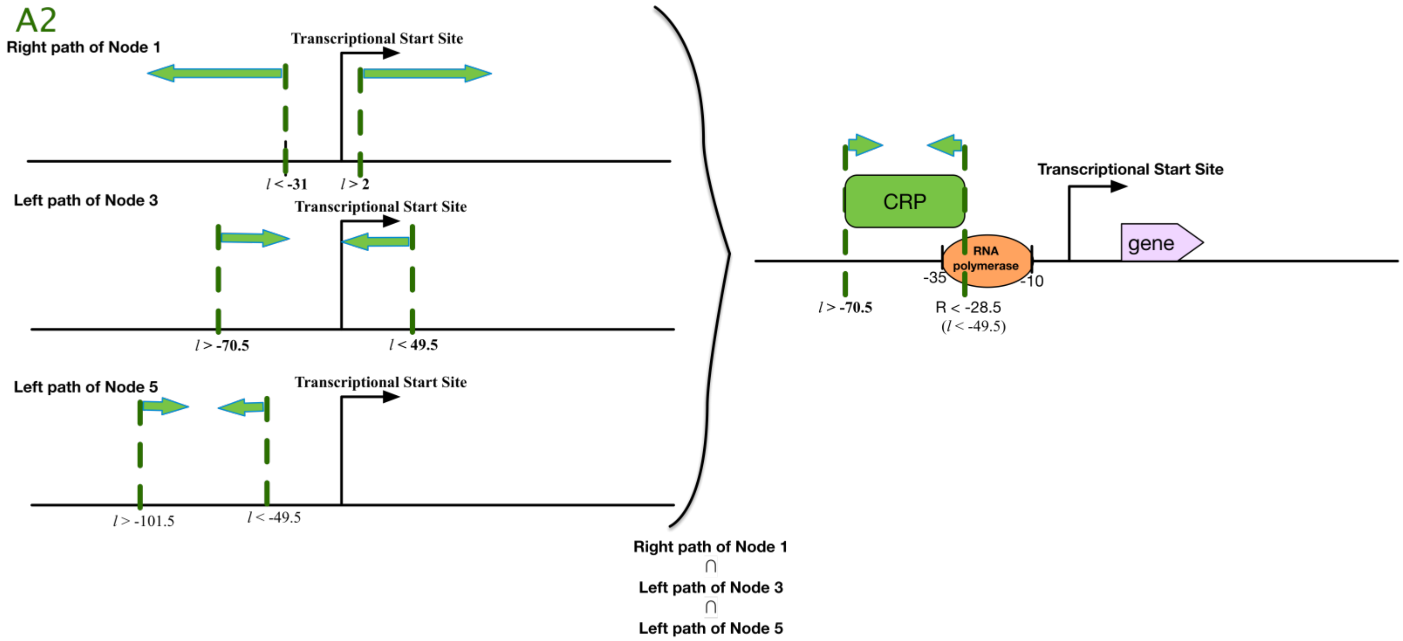

Figure S3. The inference process of the location critieria of activation rule 2.

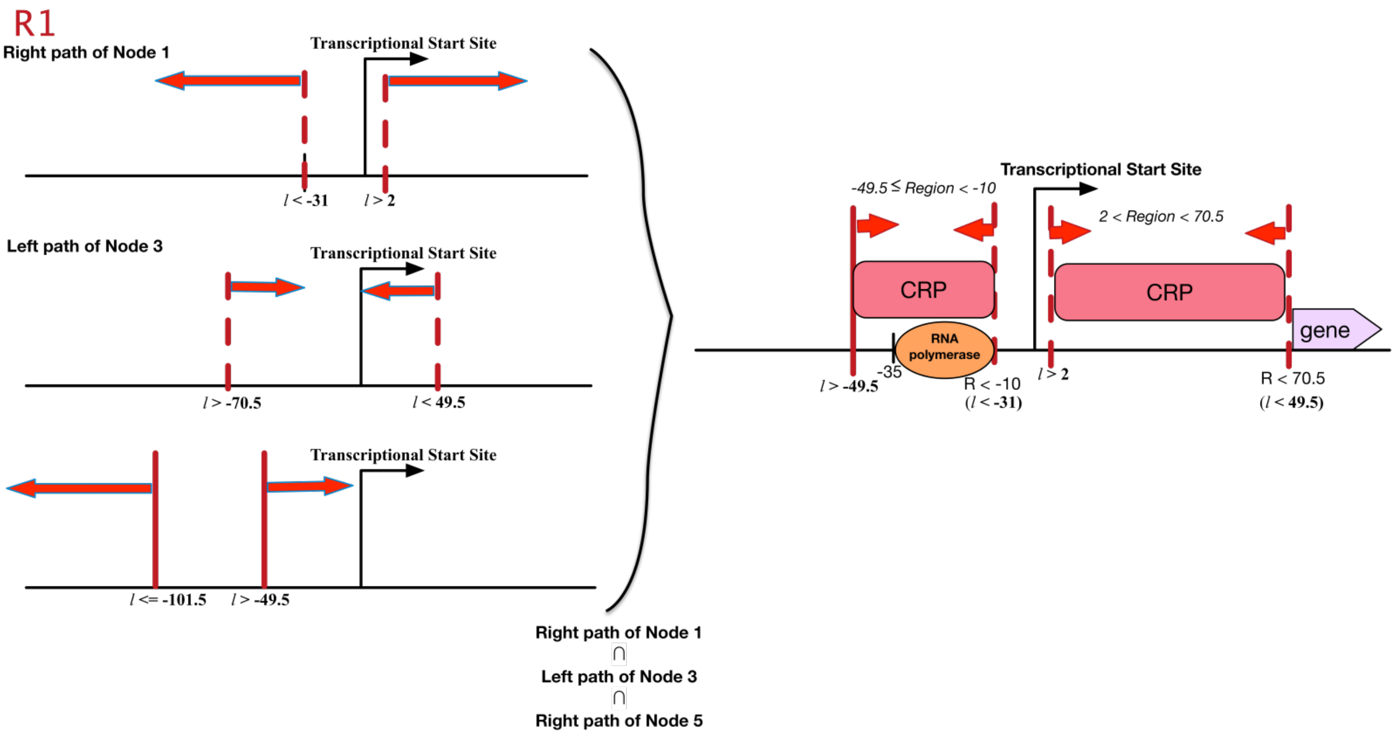

Figure S4. The inference process of the location critieria of repression rule 1.

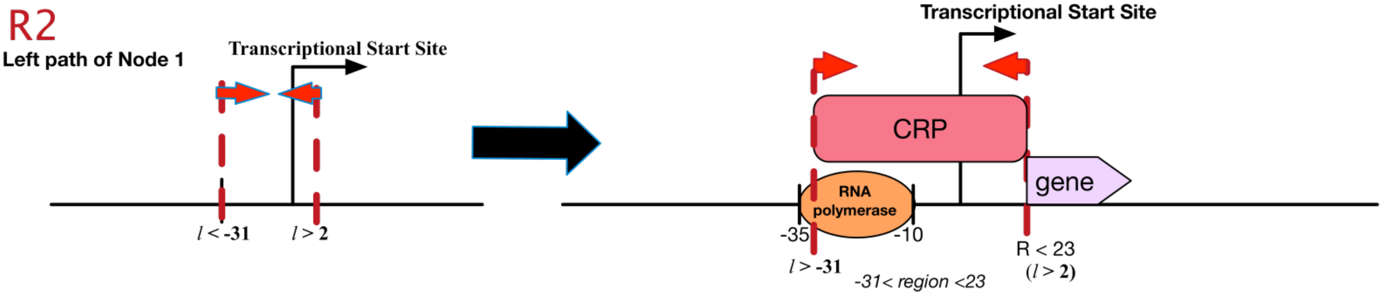

Figure S5. The inference process of the location critieria of repression rule 2.

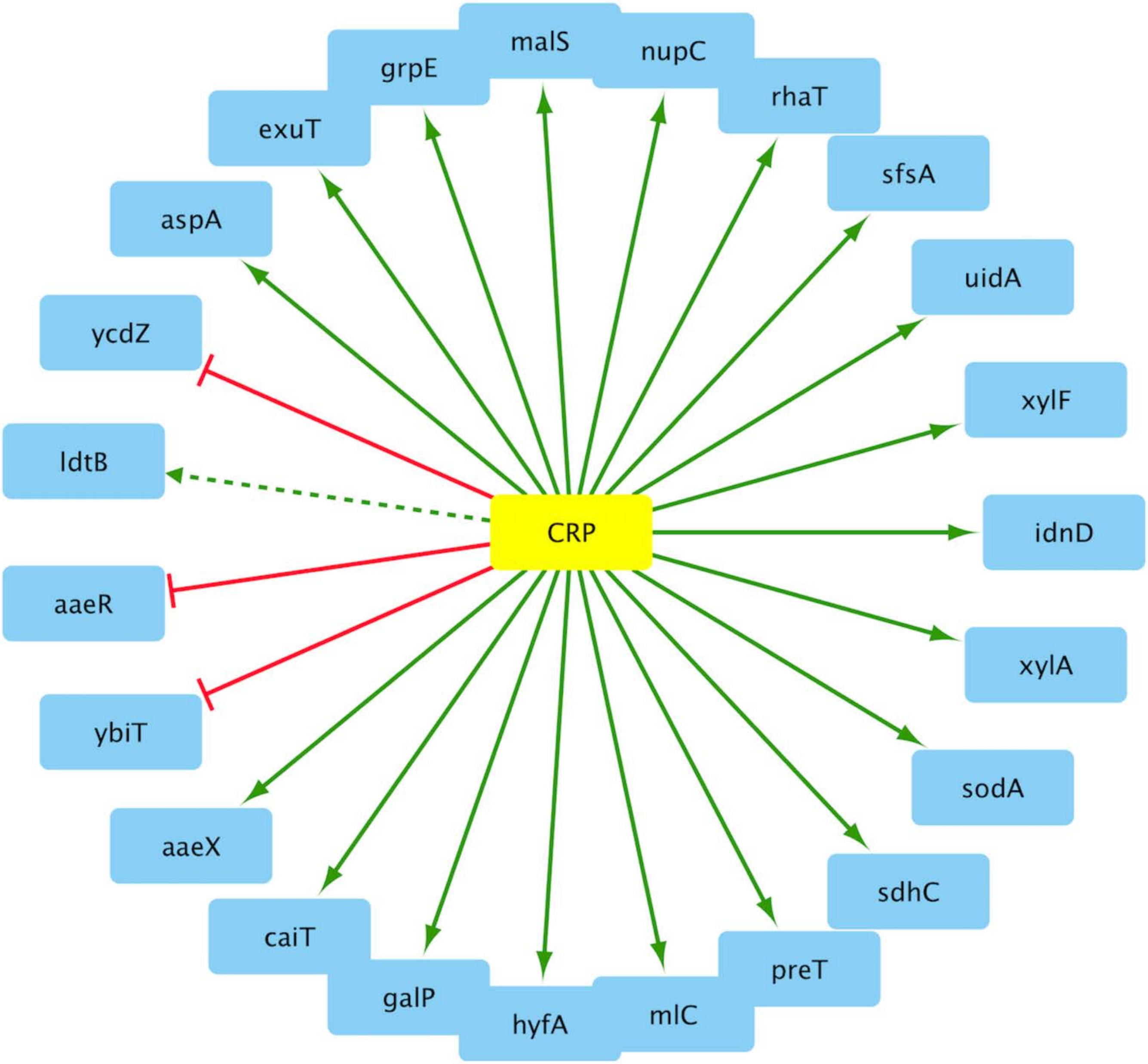

Figure S6. The CRP-regulated interactions where the regulatory roles were predicted by PredCRP.

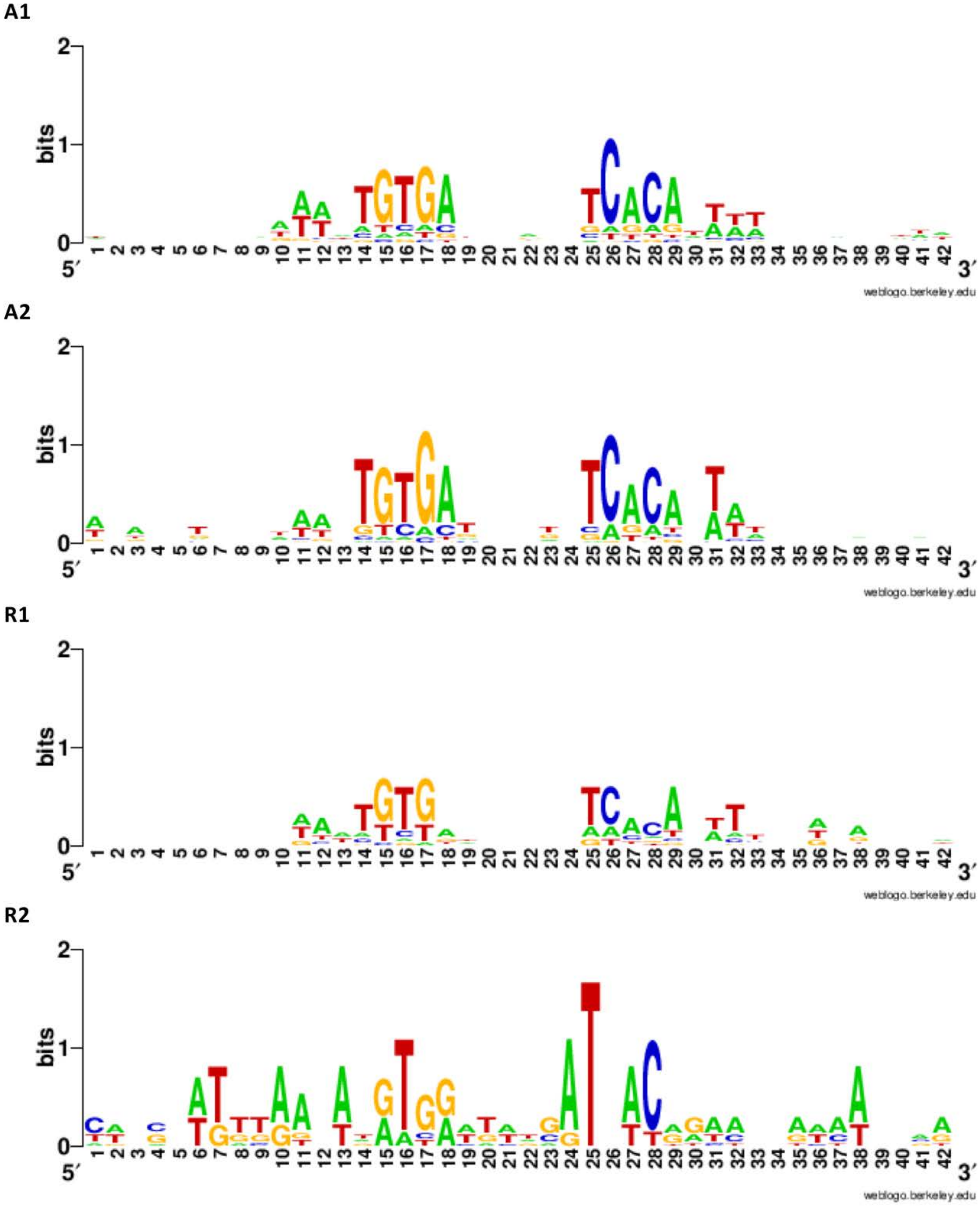

Figure S7. Sequence logo of each rule obtained from WebLogo

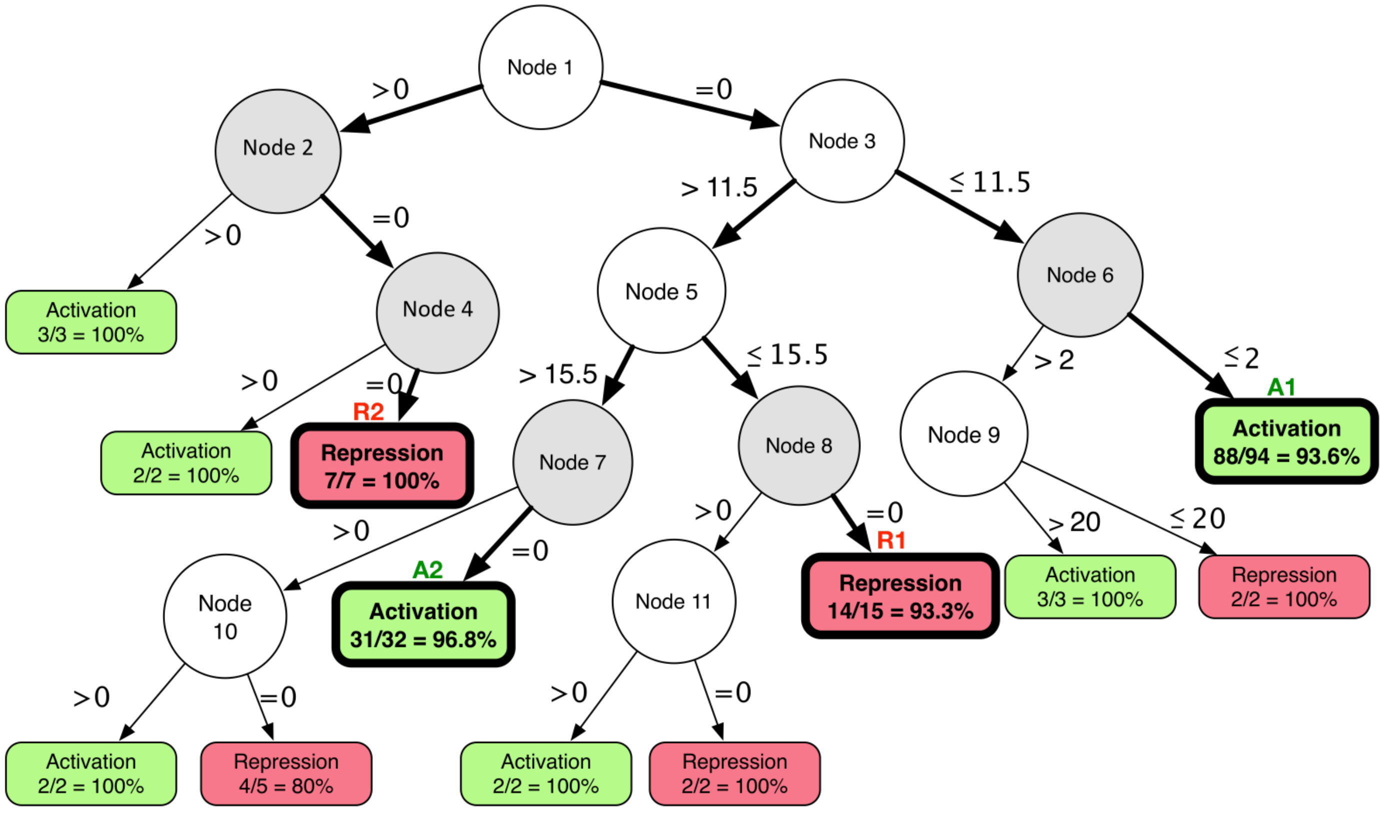

Figure S8. The nodes of the decision tree are numbered in both top-to-down and left-to-right manners.

The gray nodes are related to informative motifs. On the other hand, the white ones are related to locations of CRP-binding sites.

References

1 Savery, N. J. et al. Transcription activation at Class II CRP-dependent promoters: identification of determinants in the C-terminal domain of the RNA polymerase alpha subunit. *The EMBO journal* **17**, 3439-3447; DOI:10.1093/emboj17.12.3439 (1998).

2 Zheng, D., Constantinidou, C., Hobman, J. L. & Minchin, S. D. Identification of the CRP regulon using in vitro and in vivo transcriptional profiling. *Nucleic Acids Res.* **32**, 5874-5893; DOI:10.1093/nar/gkh908 (2004).

3 Busby, S. & Ebright, R. H. Transcription activation by catabolite activator protein (CAP). *Journal of molecular biology* **293**, 199-213; DOI:10.1006/jmbi.1999.3161 (1999).

4 Chen, Y. P., Lin, H. H., Yang, C. D., Huang, S. H. & Tseng, C. P. Regulatory role of cAMP receptor protein over Escherichia coli fumarase genes. *J Microbiol*. **50**, 426-433; DOI:10.1007/s12275-012-1542-6 (2012).

5 Yang, C. D., Chen, Y. H., Huang, H. Y., Huang, H. D. & Tseng, C. P. CRP represses the CRISPR/Cas system in Escherichia coli: evidence that endogenous CRISPR spacers impede phage P1 replication. *Mol. Microbiol.* **92**, 1072-1091; DOI:10.1111/mmi.12614 (2014).

6 Keseler, I. M. et al. The EcoCyc database: reflecting new knowledge about Escherichia coli K-12. *Nucleic Acids Res.* **45**, D543-D550; DOI:10.1093/nar/gkw1003 (2017).

7 Huang, W. L., Tung, C. W., Liaw, C., Huang, H. L. & Ho, S. Y. Rule-Based Knowledge Acquisition Method for Promoter Prediction in Human and Drosophila Species. *Scientific World Journal* (2014).

8 van Hijum, S. A. F. T., Medema, M. H. & Kuipers, O. P. Mechanisms and Evolution of Control Logic in Prokaryotic Transcriptional Regulation. *Microbiology and Molecular Biology Reviews* **73**, 481 (2009).

9 Huang, W. L., Tung, C. W., Huang, H. L., Hwang, S. F. & Ho, S. Y. ProLoc: Prediction of protein subnuclear localization using SVM with automatic selection from physicochemical composition features. *Biosystems* **90**, 573-581 (2007).

10 Yang, J. Y., Zhou, Y., Yu, Z. G., Anh, V. & Zhou, L. Q. Human Pol II promoter recognition based on primary sequences and free energy of dinucleotides. *BMC Bioinformatics* **9** (2008).

11 Zhang, Z. D., Kochhar, S. & Grigorov, M. G. Descriptor-based protein remote homology identification. *Protein Science* **14**, 431-444 (2005).

12 Chang, C. C. & Lin, C. J. LIBSVM: A Library for Support Vector Machines. *ACM Transactions on Intelligent Systems and Technology* **2** (2011).

13 Kozera, B. & Rapacz, M. Reference genes in real-time PCR. *J Appl. Genet.* **54**, 391-406; DOI:10.1007/s13353-013-0173-x (2013).

14 Raghavan, R., Sage, A. & Ochman, H. Genome-Wide Identification of Transcription Start Sites Yields a Novel Thermosensing RNA and New Cyclic AMP Receptor Protein-Regulated Genes in Escherichia coli. *Journal of Bacteriology* **193**, 2871-2874; DOI:10.1128/jb.00398-11 (2011).

15 Zheng, D. L., Constantinidou, C., Hobman, J. L. & Minchin, S. D. Identification of the CRP regulon using in vitro and in vivo transcriptional profiling. *Nucleic Acids Res.* **32**, 5874-5893; DOI:10.1093/nar/gkh908 (2004).

16 Sernova, N. V. & Gelfand, M. S. Comparative genomics of CytR, an unusual member of the LacI family of transcription factors. *PLoS One* **7**, e44194; DOI:10.1371/journal.pone.0044194 (2012).

17 Tsunedomi, R., Izu, H., Kawai, T. & Yamada, M. Dual control by regulators, GntH and GntR, of the GntII genes for gluconate metabolism in Escherichia coli. *J. Mol. Microbiol. Biotechnol.* **6**, 41-56; DOI:73407 (2003).

18 Golby, P., Kelly, D. J., Guest, J. R. & Andrews, S. C. Transcriptional regulation and organization of the dcuA and dcuB genes, encoding homologous anaerobic C4-dicarboxylate transporters in Escherichia coli. *J. Bacteriol*. **180**, 6586-6596 (1998).

19 Gosset, G., Zhang, Z., Nayyar, S., Cuevas, W. A. & Saier, M. H., Jr. Transcriptome analysis of Crp-dependent catabolite control of gene expression in Escherichia coli. *J Bacteriol.* **186**, 3516-3524; DOI:10.1128/JB.186.11.3516-3524.2004 (2004).

20 Buchet, A., Eichler, K. & Mandrand-Berthelot, M. A. Regulation of the carnitine pathway in Escherichia coli: investigation of the cai-fix divergent promoter region. *J. Bacteriol.* **180**, 2599-2608 (1998).

21 Buchet, A., Nasser, W., Eichler, K. & Mandrand-Berthelot, M. A. Positive co-regulation of the Escherichia coli carnitine pathway cai and fix operons by CRP and the CaiF activator. *Mol. Microbiol.* **34**, 562-575 (1999).

22 Eichler, K., Bourgis, F., Buchet, A., Kleber, H. P. & Mandrand-Berthelot, M. A. Molecular characterization of the cai operon necessary for carnitine metabolism in Escherichia coli. *Mol. Microbiol.* **13**, 775-786 (1994).

23 Rodionov, D. A., Mironov, A. A., Rakhmaninova, A. B. & Gelfand, M. S. Transcriptional regulation of transport and utilization systems for hexuronides, hexuronates and hexonates in gamma purple bacteria. *Mol. Microbiol.* **38**, 673-683 (2000).

24 Weickert, M. J. & Adhya, S. The galactose regulon of Escherichia coli. *Mol. Microbiol.* **10**, 245-251 (1993).

25 Self, W. T., Hasona, A. & Shanmugam, K. T. Expression and regulation of a silent operon, hyf, coding for hydrogenase 4 isoenzyme in Escherichia coli. *J. Bacteriol.* **186**, 580-587 (2004).

26 Schneider, E., Freundlieb, S., Tapio, S. & Boos, W. Molecular characterization of the MalT-dependent periplasmic alpha-amylase of Escherichia coli encoded by malS. *J. Biol. Chem.* **267**, 5148-5154 (1992).

27 Otsuka, J., Watanabe, H. & Mori, K. T. Evolution of transcriptional regulation system through promiscuous coupling of regulatory proteins with operons; suggestion from protein sequence similarities in Escherichia coli. *J. Theo.r Biol.* **178**, 183-204 (1996).

28 Shin, D., Lim, S., Seok, Y. J. & Ryu, S. Heat shock RNA polymerase (E sigma(32)) is involved in the transcription of mlc and crucial for induction of the Mlc regulon by glucose in Escherichia coli. *J. Biol. Chem.* **276**, 25871-25875; DOI:10.1074/jbc.M101757200 (2001).

29 Craig, J. E., Zhang, Y. & Gallagher, M. P. Cloning of the nupC gene of Escherichia coli encoding a nucleoside transport system, and identification of an adjacent insertion element, IS 186. *Mol. Microbiol.* **11**, 1159-1168 (1994).

30 Valentin-Hansen, P. et al. Design of cAMP-CRP-activated promoters in Escherichia coli. *Mol. Microbiol.* **5**, 433-437 (1991).

31 Mihara, H., Hidese, R., Yamane, M., Kurihara, T. & Esaki, N. The iscS gene deficiency affects the expression of pyrimidine metabolism genes. *Biochem. Biophys. Res. Commun. 372*, 407-411; DOI:10.1016/j.bbrc.2008.05.019 (2008).

32 Via, P., Badia, J., Baldoma, L., Obradors, N. & Aguilar, J. Transcriptional regulation of the Escherichia coli rhaT gene. *Microbiology* **142** (Pt 7), 1833-1840;DOI:10.1099/13500872-142-7-1833 (1996).

33 Zhang, Z. et al. Functional interactions between the carbon and iron utilization regulators, Crp and Fur, in Escherichia coli. *J. Bacteriol.* **187**, 980-990;DOI:10.1128/JB.187.3.980-990.2005 (2005).

34 Kawamukai, M. et al. Nucleotide sequence and characterization of the sfs1 gene: sfs1 is involved in CRP-dependent mal gene expression in Escherichia coli. *J. Bacteriol.* **173**, 2644-2648 (1991).

35 Blanco, C., Mata-Gilsinger, M. & Ritzenthaler, P. The use of gene fusions to study the expression of uidR, a negative regulatory gene of Escherichia coli K-12. *Gene* **36**, 159-167 (1985).

36 Song, S. & Park, C. Organization and regulation of the D-xylose operons in Escherichia coli K-12: XylR acts as a transcriptional activator. *J. Bacteriol.* **179**, 7025-7032 (1997).
